# RETRACTED: Shen et al. Cold-Pressed *Nigella Sativa* Oil Standardized to 3% Thymoquinone Potentiates Omega-3 Protection against Obesity-Induced Oxidative Stress, Inflammation, and Markers of Insulin Resistance Accompanied with Conversion of White to Beige Fat in Mice. *Antioxidants* 2020, *9*, 489

**DOI:** 10.3390/antiox14070758

**Published:** 2025-06-20

**Authors:** Hsin Hsueh Shen, Stephen J. Peterson, Lars Bellner, Abu Choudhary, Lior Levy, Leah Gancz, Ariel Sasson, Joseph Trainer, Rita Rezzani, Abraham Resnick, David E. Stec, Nader G. Abraham

**Affiliations:** 1 Department of Medicine, New York Medical College, Valhalla, NY 10595, USA; hsshen@masil.ndmctsgh.edu.tw (H.H.S.); llevy6@student.nymc.edu (L.L.); lgancz@student.touro.edu (L.G.); asasson@student.nymc.edu (A.S.); jtrainer@student.nymc.edu (J.T.); abraham.resnick@touro.edu (A.R.); 2 Department and Institute of Pharmacology, National Defense Medical Center, Taipei 114, Taiwan; 3 Department of Medicine, Weill Cornell Medicine, New York, NY 10065, USA; stp9039@nyp.org; 4 New York Presbyterian Brooklyn Methodist Hospital, Brooklyn, NY 11215, USA; abc9031@nyp.org; 5 Department of Pharmacology, New York Medical College, Valhalla, New York, NY 10595, USA; lars.bellner@gmail.com; 6 Anatomy and Physiopathology Division, Department of Clinical and Experimental Sciences, University of Brescia, 25123 Brescia, Italy; rita.rezzani@unibs.it; 7 Department of Physiology and Biophysics, Cardiorenal and Metabolic Diseases Research Center, University of Mississippi Medical Center, Jackson, MS 39216, USA; 8 Joan C. Edwards School of Medicine, Marshall University, Huntington, WV 25701, USA

The journal retracts the article “Cold Press Nigella Sativa oil standardized to 3%, thymoquinone Potentiates Omega-3 Protection against Obesity-Induced Oxidative Stress, Inflammation and Insulin Resistance via Conversion of White to Beige Fat in Mice” [1] cited above.

Following publication, concerns were brought to the attention of the Editorial Office regarding duplications and indications of inappropriate image modification within the figures presented in this article [1].

Adhering to our standard procedure, an investigation was conducted by the Editorial Office and Editorial Board that confirmed a partial overlap between images in Figures 3A and 6A presenting different experimental conditions, as well as unusual image anomalies and duplication between Figures 3A and 7A. Following discussions, the authors were unable to provide satisfactory explanations, or appropriate raw material for the Editorial Board’s evaluation. Consequently, the Editorial Board has lost confidence in the reliability of the findings and has decided to retract this publication [1], as per MDPI’s retraction policy (https://www.mdpi.com/ethics#_bookmark30).

This retraction was approved by the Editor-in-Chief of the journal *Antioxidants.*

Lars Bellner agrees to this retraction. Stephen J. Peterson disagrees to this retraction. The remaining authors did not provide a comment on this decision.
